# How Did the TB Patients Reach DOTS Services in Delhi? A Study of Patient Treatment Seeking Behavior

**DOI:** 10.1371/journal.pone.0042458

**Published:** 2012-08-06

**Authors:** Sunil K. Kapoor, A. Venkat Raman, Kuldeep Singh Sachdeva, Srinath Satyanarayana

**Affiliations:** 1 Harrow Medical Centre, Noida, India; 2 Faculty of Management Studies, University of Delhi, New Delhi, India; 3 Central TB Division, Directorate General of Health Services, Ministry of Health and Family Welfare, Govt. of India, New Delhi, India; 4 International Union Against Tuberculosis and Lung Disease, South-East Asia Regional Office, New Delhi, India; Institut de Pharmacologie et de Biologie Structurale, France

## Abstract

**Setting:**

Revised National Tuberculosis Control Programme (RNTCP), Delhi, India.

**Objective:**

To ascertain the number and sequence of providers visited by TB patients before availing treatment services from DOTS; to describe the duration between onset of symptoms to treatment.

**Study design:**

A cross sectional, qualitative study. Information was gathered through in-depth interviews of TB patients registered during the month of Oct, 2012 for availing TB treatment under the Revised National TB Control Programme from four tuberculosis diagnosis and treatment centers in Delhi.

**Results:**

Out of the 114 patients who registered, 108 participated in the study. The study showed that informal providers and retail chemists were the first point of contact and source of clinical advice for two-third of the patients, while the rest sought medical care from qualified providers directly. Most patients sought medical care from more than two providers, before being diagnosed as TB. Female TB patients and patients with extra-pulmonary TB had long mean duration between onset of symptoms to initiation of treatment (6.3 months and 8.4 months respectively).

**Conclusion:**

The pathways followed by TB patients, illustrated in this study, provide valuable lessons on the importance of different types of providers (both formal and informal) in the health system in a society like India and the delays in the diagnosis and treatment of tuberculosis.

## Introduction

Globally, the burden of Tuberculosis (TB) is estimated to be highest in India. In 2010, out of the estimated global annual incidence of 8.8 million TB cases, nearly 2.2 million cases have been reported in India, of whom 0.9 million were infectious cases [1]. Success of TB control depends on early diagnosis and appropriate treatment of TB cases and is the epidemiological basis of the global plan to stop TB [2].

Evidence suggests that TB patients, particularly from low income groups or underserved areas take long and sometimes difficult pathways to reach health facilities that provide appropriate care [3]. However, such studies provide little evidence in terms of tracking and mapping the pathways taken by TB patients, to reach appropriate treatment facilities.

Under the Revised National Tuberculosis Control Programme (RNTCP) of the Government of India, provisions have been made to provide access to diagnostic and treatment services in a decentralized manner through a network of ∼13,000 sputum smear microscopy centers and ∼650,000 of Directly Observed Treatment Short-course (DOTS) centers within the public health system [4].

In India, health care is predominantly sought from the private sector [5]. A large majority of the private sector are individual providers, who are both formal (qualified) and informal (non-qualified) providers [6], [7]. The poor and socially marginalized communities usually seek health services from informal providers [8], [9]. Symptoms of TB are such that patients often mistake it for routine illness and tend to seek first clinical advice either from a retail chemist or an informal provider - as they would do for other illnesses. Temporary relief followed by recurrence of same symptoms, drives the patient to seek clinical opinion once again from the same provider or other similar providers. Confounded by persistent symptoms without any relief from a number of providers, the patients may finally turn up at the appropriate health facility (like DOTS center) at a later stage of their disease [10]. Reports suggest even formal private providers indulge in irrational treatment protocols, without proper diagnosis [11]. Despite anecdotal evidence on this phenomenon, there is little empirical evidence in the literature on the pathways and dynamics associated with health seeking behaviour of TB patients before they reach a formal health facility for diagnosis and treatment. It is against this background a study was undertaken with an objective to map the type of providers and the sequence of these providers visited by TB patients prior to the initiation of treatment under RNTCP. The specific objectives of the study were to: i) ascertain the number and sequence of providers visited by each patient; ii) identify the source of referrals for seeking services from different type of providers; iii) identify time gap between consultations from different providers; and iv) the extent of delay before reaching DOTS center.

## Methods

### Setting

The study was undertaken in the city of Delhi, India, which has a population of 19 million (2011 census). An estimated 40% of Delhi’s population live in slums and local clusters. Most slum dwellers are migrants from other states of India. At 209 cases per 100,000 populations per annum, Delhi is ranked among the highest rate of TB notification in the country [12]. Since 1997 (till 2009) more than 442,000 patients have been treated in the city, with a success rate of 87%. Diagnosis and treatment of TB in Delhi city is provided through a network of ∼180 DOTS centres for the diagnosis and treatment of TB. In order to avail these services, patients can directly walk in to these centers or can be referred by any of the health care providers. The study was conducted in four hospital based DOTS centers across the city- DOTS centres based in two tertiary care hospitals and two non-tertiary care hospitals. All the four facilities are located in densely populated areas. These hospitals provide health care predominantly to migrant people from slums in the vicinity. The facilities were thus chosen non-randomly.

### Study Design and Study Population

This study used qualitative research methods to gain an understanding of behavioral dynamics of the TB patients through describing and interpreting beliefs and practices while accessing medical care in a given community context. All consecutively registered TB patients in the month of October, 2010, who were availing treatment in these DOT centers, were invited to participate in the study. Patients who volunteered to participate were included in the study. Interview was conducted using a semi-structured questionnaire. Treatment records of all interviewed patients were verified from respective TB units for consistency.

### Study Instrument, Variables and Data Collection

Interviews with the patients were conducted using a pre-tested, semi-structured questionnaire by a qualified medical professional trained in qualitative research studies. The questionnaire was in first developed in English, translated into Hindi and then then back translated into English to check for consistency. The responses from the individuals was recorded verbatim, translated into English and analyzed. Patients were requested to recall and narrate their experience of visiting different providers before they sought services from DOTS Centers (facilities in the public health system for the diagnosis and treatment of TB under RNTCP). The questionnaire consisted of twenty five questions. Questions ranged from signs of early symptoms, sequence of providers visited, duration of medical care under each provider, time gap between successive providers, source of referral-if any, reasons for discontinuation of medical care, and reason to seek services from DOTS Centers. The private providers were categorized as qualified providers based on patients perception and description of the qualification of the doctor they visited (QP, doctors of allopathic system of medicine, practitioners of others systems of medicine), informal (unqualified) providers [IP, which included registered medical practitioners (RMP Doctors), Bengali Doctors etc.,], and chemists (C, pharmacy shop keepers). In addition, Social, economic and demographic information (age, gender, marital status, religion, education level, occupation, income, and family history of TB) of the responding patients were collected.

### Ethical Issues

The study was reviewed and approved from the ethics committee of the University of Delhi-South Campus. Informed written consent was obtained from each participant. In order to ensure anonymity and non-disclosure of individual information, name of the patient was not sought in the questionnaire but compiled separately using serial numbers. Confidentiality clause was readout in the local language (Hindi) and explained. In case if the participant was aged less than 18 years, additional informed written consent was obtained from the parent or guardian accompanying the patient.

### Data Entry and Analysis

Data was analyzed using Microsoft excel. Social and demographic profile of the patients was tabulated using simple descriptive statistics. Sequential movement (pathways) of patients seeking services from various providers was constructed using flow diagrams. Time taken from the on-set of symptoms to treatment at DOTS centers is been expressed in mean and standard deviation. Some of the relevant responses of the participants have been provided verbatim (after translation into English).

## Results

Out of the 114 patients registered for treatment, 108 patients [median age 40 years, range 15–60 years] agreed to participate in the study. The Profile of participants and the duration between onset of symptoms to treatment is given in Table 1. Less than a third of the patients (29%) had sought medical care within the first month from the onset of TB symptoms. Female patients had sought care later (6.3 months) when compared to men (3.8 months). People in the lowest income quartile took longer time to seek medical care (5.8 months) when compared to the people in the other quintiles. Extra pulmonary patients on an average took more than 8 months (8.4 months); Those who sought services from qualified practitioner as the first source of medical care took longer time (5.2 months) to get diagnosed as TB and reach DOTS centers compared to informal providers (4.8 months) or chemists (3.1 months).

**Table 1:** Demographic Characteristics and mean duration of from onset of symptoms to reaching DOTS Centers, Delhi, India.

Characteristics	N	%	Mean duration*	(Standard deviation)
**Sex**				
Male	60	55.6	3.8 m	(2.5)
Female	48	44.4	6.3 m	(5.1)
**Age**				
15–24 years	36	33.3	4.9 m	(4.3)
25–34 years	29	26.9	5.9 m	(4.6)
35–44 years	14	12.9	3.7 m	(3.4)
45–60 years	29	26.9	4.5 m	(3.5)
**Income (per month)**				
≤ Rs 3000	8	7.4	5.8 m	(3.4)
Rs 3001– Rs 6000	37	34.3	5.4 m	(4.2)
Rs 6001– Rs 10000	35	32.4	4.4 m	(3.6)
≥ Rs 10001	28	25.9	4.6m	(4.6)
**Type of TB**				
New Pulmonary	78	72.2	4.0 m	(2.9)
New Extra-Pulmonary	21	19.5	8.4 m	(6.2)
Relapse	9	8.3	4.6 m	(2.3)
**Source of first contact for medical care after onset of symptoms**				
Chemist	8	7.4	3.1 m	(2.2)
Informal practitioner	67	62.0	4.8 m	(5.5)
Qualified practitioner	33	30.6	5.2 m	(3.4)
**No. of health care providers consulted prior to reaching health facilities with RNTCP services**				
1	13	12.0	3.4 m	(3.5)
2	29	26.9	5.7 m	(5.5)
3	24	22.2	4.7 m	(3.7)
4	27	25.0	4.2 m	(2.1)
5	11	10.2	5.2 m	(3.5)
6	4	3.7	9.4 m	(6.3)

* mean duration in months from the onset of symptoms.

**Figure 1:**
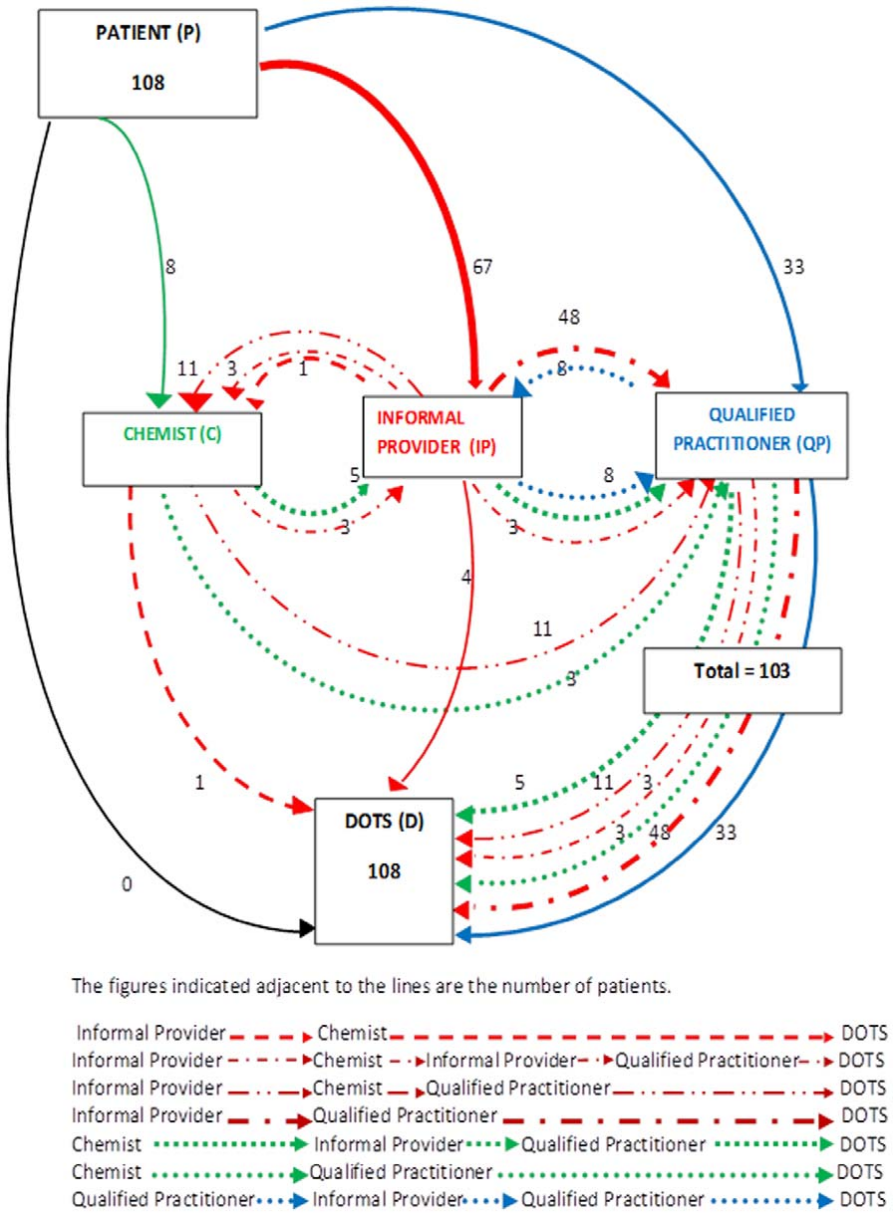
Pathways undertaken by the patients to reach the RNTCP (DOTS) Facilities, Delhi, India.

Figure 1 illustrates the pathways taken by the patients while seeking medical care from different providers. None of the patients had directly walked in to these centers seeking medical care. Nearly two thirds of the patients (n = 67) sought initial contact from informal providers as the first source of medical care, whereas less than one-third of patients (n = 33) sought services from qualified providers. The reason quoted by patients as to why they initially sought care from these informal providers is (translated into English) “we always go to Dr XX (also called locally as Bengali doctor) and recover quickly from our illnesses. He is wise and always has lot of patients in his clinic.” Not satisfied with the medical care from informal providers most (48 out of 67) then turn to qualified practitioners [patient quote “after taking medicines and injections for three weeks, I felt that this practitioner is not being able to diagnose my disease properly because he is not a qualified practitioner, so I went to a qualified (MBBS) practitioner”]. Some of those who sought services from informal providers (as the first source of medical care) also sought second opinion from either chemists or other informal providers repeatedly, before going to a qualified practitioner [the reason why chemists were visited in patient quote “these doctors charge a lot. Since all prescriptions go to a chemist, they know everything about the disease. Besides they do not charge any consultation fee. They (the chemists) have been serving our purpose well”]. Even after seeking medical care from qualified practitioner, some (n = 8) went back to informal providers. Almost all patients (n = 103) finally sought medical care from qualified practitioners, most of whom were in the private sector, before being referred to a DOTS center.

**Figure 2:**
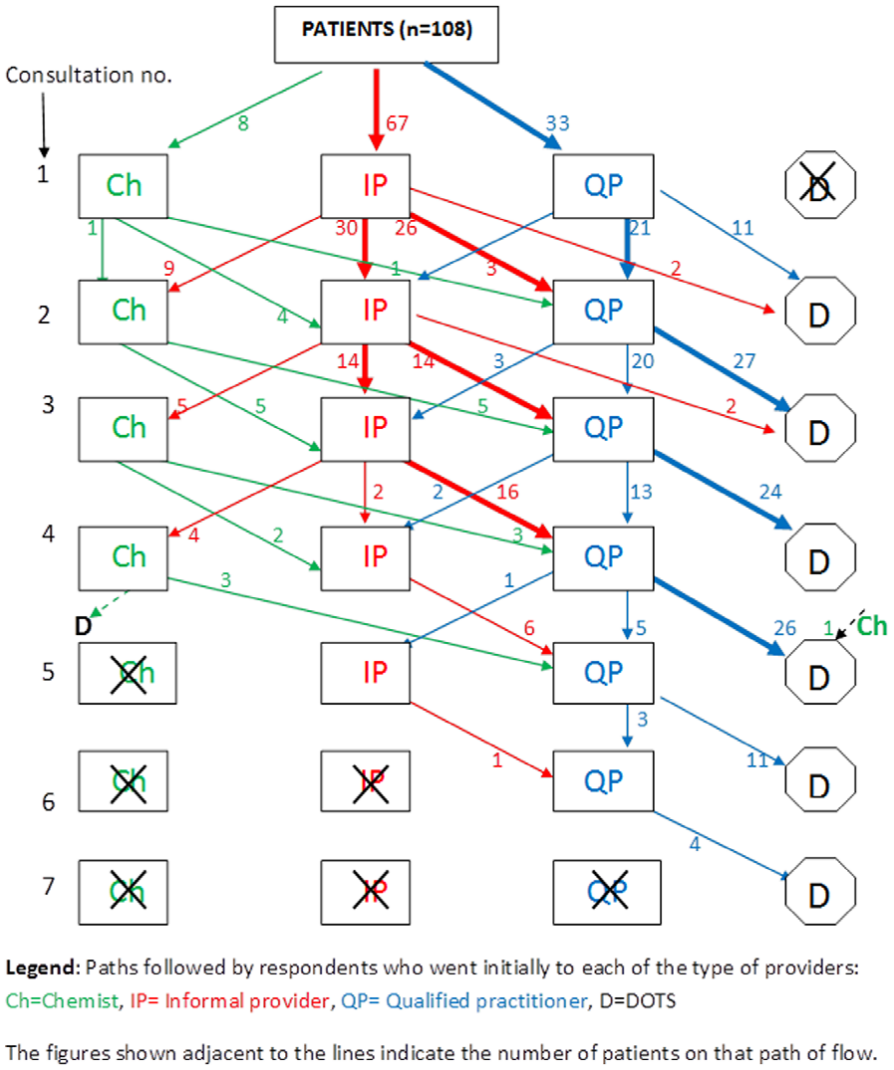
Pathway taken by patients to reach health facilities with RNTCP (DOTS) services in relation to the type of health care provider and frequency of consultation.

Figure 2, illustrates the sequence of consultations held by the patients with various providers. Those who sought medical care from chemists finally went to qualified practitioner, but only after four rounds of consultations with informal providers or other chemists. Majority of the patients despite visiting the informal providers, moved to qualified practitioners eventually. This was apparent from the proportion of patients who had sought care from qualified practitioners after the third round of consultation with the informal providers. One-third of the patients who sought medical care from qualified providers went to DOTS directly after the 1^st^ consultation, but others had visited qualified providers few more times (up to 6 times) before reaching a DOTS center.

## Discussion

In this study, an attempt has been made to trace the pathways taken by TB patients while seeking medical care for their illness. The findings of the study indicate the range and sequence of providers, duration of medical care and thus explain the possible cause of delay in diagnosis and treatment of TB. If the pathways of treatment in a city with relatively higher mean income, literacy, and easy access to health facilities are of any indication, the situation in hinterland could be worrisome. This is particularly important as India is aiming to achieve ‘Universal Access’ for quality assured diagnosis and treatment under RNTCP [13]. There are two important implications of this study finding to TB control in India.

First, it is inevitable but to recognize the importance of informal providers and chemists in the whole sequence of TB diagnosis and treatment cycle. This has been identified in other settings as well [14]–[17]. Appropriate strategies need to be evolved for the involvement of these informal providers - keeping in view the legal and technical aspects of TB care, so as to widen the coverage of RNTCP services and ensure early diagnosis of TB. It would be futile if they are ignored simply as ‘quacks’ as they are likely to exist in the Indian context in the foreseeable future. There is a need to recognize them as one of the key stakeholders in any public-private mix (PPM) strategy. Experience of pilot initiatives involving informal providers in TB care in some Indian states like Bihar has been encouraging [4] and initiatives are being undertaken in for their involvement across the country [18].

Second, success of TB control is possible only if the illness is diagnosed and treated in time, irrespective of the place of diagnosis or treatment. However, delays caused due to complex pathways followed by the TB patients before they reach a DOTS Center- as illustrated in this study, not only jeopardize their own wellbeing but also makes others in the community vulnerable for infection. Current Information Education and Communication and/or Behavior Change Communication strategies need to incorporate messages highlighting the need for patients to visit DOTS center, if they don’t get relief from symptoms even though they are visiting health care providers that are known to them.

### Limitation

First, as in all observational studies, which elicit information on past experiences of the patients, this study may have recall bias of patients. But efforts were made to minimize errors in recall by asking them to relate with events, dates and co-relating it to available medical records. Second, the study was conducted in a few selected sites by recruiting patients registered during one month. Hence the study findings cannot be generalized to outside the study sites and the studied participants. However, the study finding does provide guidance on the potential pathways taken by patients. Third, the classification of providers into formal and informal was based on the perception and description of the patients, and we did not attempt to cross verify the correctness of this information by visiting the health care providers as this was not operationally feasible.

### Conclusion

The pathways followed by TB patients, illustrated in this study, shows the care seeking behavior from different types of providers (formal and informal) in the health system in a society like India and the duration between onset of symptoms to treatment of TB. There is a need to incorporate measures to address these issues in the TB control strategy of the country.

## References

[1] World Health Organisation, Geneva (2012) Global tuberculosis control 2011. Available: http://www.who.int/tb/publications/global_report/en/index.html. Accessed on 2012 May 12..

[2] Stop TB Department (2011) The Global Plan to Stop TB 2011–2015, World Health Organisation, Geneva. Available: http://www.stoptb.org/global/plan/. Accessed on 2012 May 12..

[3] Sreeramareddy CT, Panduru KV, Menten J, Van den Ende J (2009) Time delays in diagnosis of pulmonary tuberculosis: a systematic review of literature. BMC Infect Dis 9: 91. 1471-2334-9-91 [pii];10.1186/1471-2334-9-91 [doi]. 10.1186/1471-2334-9-91 PMC2702369 19519917

[4] Central Tuberculosis Division (2011) Tuberculosis India 2011, Annual report of the Revised National Tuberculosis Control Programme, Ministry of Health and Family Welfare, Government of India. Available: http://www.tbcindia.nic.in/pdfs/RNTCP%20TB%20India%202011.pdf. Accessed on 2012 May 19..

[5] Uplekar MW (2003) Public-private mix for DOTS: demanding, but delay will only hamper TB control. Int J Tuberc Lung Dis 7: 1113–1114. 14677884

[6] Price Waterhouse Coopers (2007) Health Care in India. Emerging Market Report. Available: http://www.pwc.com/en_GX/gx/healthcare/pdf/emerging-market-report-hc-in-india.pdf. Accessed on 2010 May 15..

[7] Bajpai V, Saraya A (2010) Healthcare financing: approaches and trends in India. Natl Med J India 23: 231–235. 21192520

[8] Yesudian CAK (2011) Health seeking behaviour of urban poor in India. Available: http://www.hum.au.dk/hsre/Docs/Presentations/2_Private%20health%20sector/B.Health%20Seeking%20Behaviour/1_CAK_Yesudian.pdf. Accessed on 2011 Jun 5..

[9] A.Venkat Raman, James Warner Björkman (2008) Public Private Pratnerships in Health Care in India. Lessons for Developing Countries. Available: http://www.routledge.com/books/details/9780415467285/. Accessed on 2012 June 30..

[10] Charles N, Thomas B, Watson B, Raja SM, Chandrasekeran V, Wares F (2010) Care seeking behavior of chest symptomatics: a community based study done in South India after the implementation of the RNTCP. PLoS One 5 10.1371/journal.pone.0012379 [doi]. 10.1371/journal.pone.0012379 PMC2942833 20862219

[11] Udwadia ZF, Pinto LM, Uplekar MW (2010) Tuberculosis management by private practitioners in Mumbai, India: has anything changed in two decades? PLoS One 5: e12023 10.1371/journal.pone.0012023 [doi]. 20711502 10.1371/journal.pone.0012023 PMC2918510

[12] Delhi State TB Cell (2011) DOTS services in Delhi. Available: http://www.dotsdelhi.org/program-performance.php. Accessed on 2011 Jun 4..

[13] Central TB Division (2010) Universal access to TB Care- A practical guide for programme managers. Directorate General of Health Services, Ministry of Health and Family Welfare, Government of India. Available: http://www.tbcindia.nic.in/pdfs/Universal_accessto_TB_Care.pdf. Accessed on 2012 May 12..

[14] Sheikh K, Porter J, Kielmann K, Rangan S (2006) Public-private partnerships for equity of access to care for tuberculosis and HIV/AIDS: lessons from Pune, India. Trans R Soc Trop Med Hyg 100: 312–320. S0035-9203(05)00384-6 [pii];10.1016/j.trstmh.2005.04.023 [doi]. 16438997 10.1016/j.trstmh.2005.04.023

[15] Annapurna Chavali (2010) Informal provision of health care- A look at the Indian Context. Available: http://healthmarketinnovations.org/blog/2010/nov/17/informal-provision-health-care. Accessed on 2012 May 30..

[16] Gautham M, Binnendijk E, Koren R, Dror DM (2011) ‘First we go to the small doctor’: first contact for curative health care sought by rural communities in Andhra Pradesh & Orissa, India. Indian J Med Res 134: 627–638. IndianJMedRes_2011_134_5_627_90987 [pii]; 10.4103/0971-5916.90987 [doi]. 22199101 10.4103/0971-5916.90987 PMC3249960

[17] Mignone J, Washington RG, Ramesh BM, Blanchard JF, Moses S (2007) Formal and informal sector health providers in southern India: role in the prevention and care of sexually transmitted infections, including HIV/AIDS. AIDS Care 19: 152–158. 769829767 [pii];10.1080/09540120600780542 [doi]. 17364393 10.1080/09540120600780542

[18] The Union South-East Asia Regional Office, New Delhi (2010) Project Axshya: A Global Fund Round 9 Grant supported Advocacy, Communication and Social Mobilisation Project for TB care and Control in India. Available: http://www.axshya-theunion.org/. Accessed on 2012 Mar 12..

